# Killing Two Birds with One Stone: Potential Therapies Targeting Psoriasis and Atherosclerosis at the Same Time

**DOI:** 10.3390/ijms23126648

**Published:** 2022-06-14

**Authors:** Eva Klara Merzel Šabović, Mateja Starbek Zorko, Miodrag Janić

**Affiliations:** 1 Department of Dermatovenerology, University Medical Centre Ljubljana, Gradiškova Ulica 10, SI-1000 Ljubljana, Slovenia; mateja.starbekzorko@kclj.si; 2 Faculty of Medicine, University of Ljubljana, Vrazov Trg 2, SI-1000 Ljubljana, Slovenia; miodrag.janic@kclj.si; 3 Department of Endocrinology, Diabetes and Metabolic Diseases, University Medical Centre Ljubljana, Zaloška Cesta 7, SI-1000 Ljubljana, Slovenia

**Keywords:** psoriasis, cardiometabolic risk, atherosclerosis, antipsoriatic treatment, cardiometabolic treatment

## Abstract

Psoriasis is a chronic systemic inflammatory disease. Due to systemic inflammation, it is associated with many comorbidities. Among them, cardiovascular diseases represent the most common causes of morbidity and mortality in this population. Therefore, physicians treating patients with psoriasis should keep in mind that, as important as the treatment of psoriasis, awareness of cardiovascular risk deserves additional attention. Thus, in parallel with psoriasis treatment, a cardiovascular risk assessment must also be performed and addressed accordingly. In addition to encouraging non-pharmacologic strategies for a healthy lifestyle, physicians should be familiar with different pharmacologic options that can target psoriasis and reduce cardiovascular risk. In the present article, we present the pathophysiological mechanisms of the psoriasis and cardiometabolic interplay, our view on the interaction of psoriasis and cardiovascular disease, review the atherosclerotic effect of therapeutic options used in psoriasis, and vice versa, i.e., what the effect of medications used in the prevention of atherosclerosis could be on psoriasis.

## 1. Introduction

Psoriasis is a chronic systemic inflammatory disease that affects approximately 2% of the global population [1]. Chronic inflammation associated with psoriasis is now known to be “more than skin deep”, that is, in addition to the skin, other organs can also be affected [2]. As is the case for most chronic inflammatory diseases, psoriatic systemic inflammation frequently affects the cardiovascular system [1,3]. Therefore, cardiovascular diseases are the leading cause of morbidity and mortality in patients with psoriasis. Atherosclerotic cardiovascular diseases decrease the life expectancy of patients with psoriasis by approximately 5 years [4]. Furthermore, traditional cardiovascular risk factors such as arterial hypertension, dyslipidemia, insulin resistance or diabetes, physical inactivity, obesity, and smoking are also frequently present in these patients, thus further increasing their cardiovascular risk [1]. However, it is now also known that psoriasis per se is an independent cardiovascular risk factor [5].

Unfortunately, data suggest that patients with psoriasis are not sufficiently screened for cardiovascular disease [6]. Furthermore, when screened, the risk of cardiovascular disease is often underestimated, according to a recent comprehensive review [7]. This is due to traditional scoring systems, which yet do not yet include chronic inflammatory diseases as an important additional cardiovascular risk factor. The most recent guidelines on the prevention of cardiovascular disease by the European Society of Cardiology recognize this problem and state that, for rheumatoid arthritis and inflammatory bowel disease, an increased risk could be estimated at 50% and 20% above the traditional calculated risk, respectively. The authors also state that in psoriasis, this level of additional risk, although undoubtedly increased, could not be adequately determined due to a lack of sufficient data [8]. 

However, awareness of this increased cardiovascular risk in patients with psoriasis appears finally to be acknowledged. The objective of this review is to convince the need for risk assessment in this patient population, as most of them have an elevated risk [6]. In addition, we provide cumulative data on antipsoriatic and anti-atherosclerotic treatment to alleviate the choice of systemic treatment in high-risk patients with psoriasis so that physicians could choose a treatment that targets both psoriasis and atherosclerosis.

## 2. Mechanistic Associations between Psoriasis and Cardiometabolic Diseases

Psoriatic march is a term that describes a series of events that lead from psoriasis to cardiovascular disease. These events include systemic inflammation, endothelial function, and insulin resistance [9].

The inciting event that leads to psoriasis is yet unknown. This event then triggers a pro-inflammatory state that promotes oxidative stress that exceeds the ability of antioxidant mechanisms. In addition, oxidative stress is also enhanced by the frequent presence of additional cardiovascular risk factors (hypertension, insulin resistance, diabetes, dyslipidemia, obesity, alcohol consumption, smoking, stress, etc.) [10]. 

Inflammation plays a central role in both the pathogenesis of psoriasis and the pathogenesis of atherosclerosis and their comorbidities. More precisely, T helper cells type 1 (Th1) and T helper cells type 17 (Th17) play crucial roles in the pathogenesis and progression of both diseases [11]. In addition, regulatory T cells that maintain immune system homeostasis by exerting anti-inflammatory characteristics are pathologically altered in both diseases that sustain overactivation of Th1 and Th17 cells [12,13]. Th1 cells secrete tumor necrosis factor-α (TNF-α) and interferon-γ (IFN-γ) which in psoriatic plaques leads to keratinocyte activation and proliferation and expression of adhesion molecules, including intercellular adhesion molecule 1 (ICAM-1) [14]. In atherosclerotic plaque, TNF- α and IFN-γ, secreted from Th1 cells, lead to plaque growth [15]. Th17 cells secrete interleukin (IL)-17 and IL-22, which in psoriatic plaque promote angiogenesis and proliferation of keratinocytes [14], while in atherosclerotic plaque they promote plaque instability by stimulating intraplaque neoangiogenesis and hemorrhage [11]. IL-17 might even weaken the fibrous cap, causing plaque rupture [16].

While the role of IL-17 in the pathogenesis of psoriasis is already well known, much less is known about the role of IL-17 in the atherosclerosis process [17]. Endothelial injury triggers the secretion of pro-inflammatory cytokines that trigger the maturation of naive T cells into a Th17 phenotype, which then transverses the junctions of endothelial cells [11]. In the arterial wall, Th17 cells secrete IL-17 which promotes the secretion of pro-inflammatory cytokines, adhesion molecules and other pro-inflammatory factors from endothelial cells, macrophages, and smooth muscle cells. In addition, endothelial and smooth muscle cells produce more IL-17, forming a harmful positive feedback loop [11,17]. Due to the deep inflammation, there is an increase in oxidative stress that exceeds the ability of antioxidant mechanisms, leading to the oxidation of low-density lipoprotein (LDL). The oxidized LDL is then phagocytosed by macrophages, transforming them into foam cells [11,18]. The oxidation of LDL in the vascular endothelium is considered an initial event in the formation of atherosclerotic plaques, because with oxidation, LDL acquires atherogenic features [18]. A newer study showed that in addition to oxidation, there could also be other modifications of LDL such as desialylation that could also increase the atherogenic potential of LDL [19]. However, oxidized LDL and its reduction still remain the most important therapeutic targets for atherosclerosis [18]. Oxidized LDL in the arterial wall promotes foam cell formation, inflammasome activation, and the expression of adhesion molecules on the cell surface, stimulating endothelial cell activation [18].

Inflammation in psoriasis is perpetuated by many factors, namely dysregulated native and adaptive immune response, increased oxidative stress, altered balance of adipokines, insulin resistance, and endothelial dysfunction [17,20,21]. Insulin resistance is commonly present in patients with psoriasis and contributes to the progression of psoriatic disease. Insulin plays an important role in the regulation of keratinocytes in psoriasis. Buerger et al. showed that tissue fluid from psoriatic patients had elevated levels of IL-1β. IL-1β activates p38 mitogen-activated protein kinases (p38MAPK), resulting in insulin resistance and disrupts insulin-dependent keratinocyte differentiation and induces keratinocyte proliferation [22]. Furthermore, inflammation-induced insulin resistance increases endothelial expression of adhesion molecules, which promotes further inflammation in psoriatic plaques [23]. Proinflammatory cytokines, whose production is markedly increased in psoriasis (TNF-α, IL-17, IL-23, and IL-6) all contribute significantly to insulin resistance in patients with psoriasis [21,24].

Chronic systemic inflammation also leads to the production of adipokines, particularly from visceral fat. Adipokine adiponectin is an insulin sensitizer that regulates glucose and lipid metabolism. Serum adiponectin level is negatively correlated with IL-6 and TNF-α levels [25]. TNF-α can decrease adiponectin secretion, which could explain lower levels of adiponectin in patients with psoriasis [26] and consequent insulin resistance [27]. Furthermore, patients with psoriasis who have metabolic syndrome, have a high body mass index, or have a higher psoriasis area and severity index (PASI) and have significantly lower adiponectin levels [28,29].

**Figure 1:**
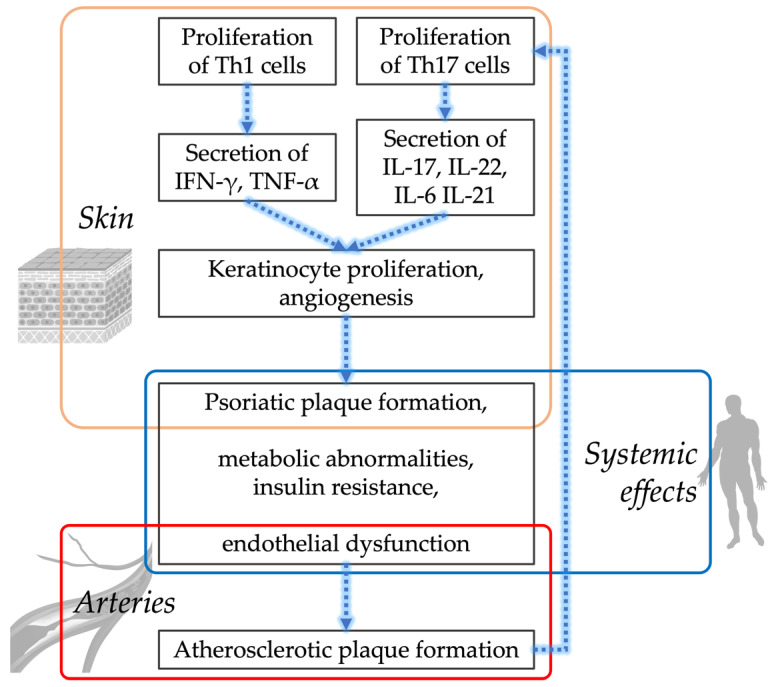
In a patient with psoriasis, there is a proliferation of Th1 and Th17 cells that secrete various cytokines. Th1 cells secrete interferon-γ (IFN-γ) and tumor necrosis factor-α (TNF-α) leading to keratinocyte activation and proliferation and expression of adhesion molecules, namely intercellular adhesion molecule-1 (ICAM-1). Th17 cells secrete interleukin (IL)-17, IL-22, IL-6, and IL-21 that stimulate keratinocyte proliferation and intraplaque angiogenesis. This systemic inflammation has systemic effects that in the skin trigger the formation of psoriatic plaques, disrupt metabolism and lead to insulin resistance and in the arteries first trigger endothelial dysfunction which then leads to the formation of atherosclerotic plaques [35].

The crucial molecule for endothelial homeostasis is nitric oxide (NO), which is produced by endothelial nitric oxide synthase (eNOS). Nitric oxide has a vasoprotective role for the endothelium. In the context of long-lasting increased oxidative stress, the functioning of eNOS can be altered [30]. eNOS is decoupled from generation of NO to generation of superoxide, which inactivates NO. This leads to endothelial dysfunction, which results in increased platelet aggregation and vasoconstriction [31]. In an animal model, IL-17A overexpression in keratinocytes was shown to induce systemic vascular inflammation, endothelial dysfunction, and arterial hypertension [17]. Another animal study showed that IL-17 induces hypertension by decreasing endothelial NO production [32]. In psoriasis, neutrophils release chemotactic agents at the site of damaged endothelium which recruit leukocytes and increase foam cells development. In addition to damaging endothelial cells, chronic inflammation through cytokines also induces insulin resistance, further affecting endothelial function [20,33]. Insulin has important direct vascular effects, namely, stimulation of endothelium NO production [20,33]. Therefore, mechanisms that contribute to insulin resistance and endothelial dysfunction (such as chronic inflammation in psoriasis) stimulate further inflammation [33]. This also explains why this harmful synergistic duo frequently co-exists in systemic inflammatory conditions, such as psoriasis. Furthermore, psoriasis with its systemic inflammation, metabolic dysfunction, and endothelial dysfunction could be considered as a continuum of psoriatic-metabolic-vascular in a patient with psoriasis (Figure 1). Consequently, each patient with psoriasis should be evaluated for their individual cardiovascular risk and additional comorbidities that could further increase this risk and treated accordingly.

Measuring endothelial function could provide important information on the systemic health status of such patients and the need for interventions. Most commonly, flow-mediated dilation, finger plethysmography, and retinal flicker test are used for measuring endothelial function [34].

## 3. Cardiometabolic Phenotype of Patients with Psoriasis 

Putting it all together, the cardiometabolic phenotype of patients with psoriasis might be as follows.

- Patients with psoriasis are frequently overweight [36];
- They frequently have arterial hypertension, which is usually difficult to control (arterial hypertension in patients with psoriasis often requires a dual or triple therapy regimen with a probability of 16.5 times higher of a triple antihypertensive regimen compared to controls) [37];
- They have dyslipidemia with an atherogenic lipid profile (hypertriglyceridemia, low high-density lipoprotein (HDL), harmful qualitative changes in low-density lipoprotein particles (LDL), and postprandial hyperlipidemia); this atherogenic lipid profile predisposes to earlier formation of atherosclerotic plaques [38];
- Due to systemic inflammation, they are also commonly affected by insulin resistance or even type 2 diabetes (prevalence of 11.6% according to a recent systematic review by Holm et al.) [39];
- Patients with psoriasis are believed to be physically less active; however, according to a recent meta-analysis, there were no significant differences in physical activity level between those with or without psoriasis, but the subgroup analysis revealed that patients with psoriasis were significantly less likely to exercise vigorously compared to controls [40]. Vigorous physical activity is independently associated with a reduced incidence of psoriasis [41]. Furthermore, patients with a larger skin area affected by psoriasis and self-awareness performed lower intensity physical activity [40]. There are also several factors that influence physical activity in a person, including obesity, gender, age, financial situation, and in the case of psoriasis, probably also the psychological impact of psoriasis and the distribution of the psoriatic lesion (visible versus nonvisible areas or functional);
- Smoking is also common among patients with psoriasis, which in addition predisposes them to accelerated cardiovascular disease. Plus, the other way around, smoking represents an independent risk factor for the development of psoriasis [42];
- Patients with psoriasis often have unbalanced diets that could influence the incidence and severity of psoriasis and its comorbidities. Their diet often has a higher intake of saturated fatty acids, noncomplex sugars, red meat, and alcohol, which further exacerbate psoriasis and its comorbidities by activating the inflammasome cascade, tumor necrosis factor-α (TNF-α)–interleukin (IL) 23–IL-17 axis, generation of reactive oxygen species, intestinal dysbiosis, and suppression of regulatory T cells [43];
- Depression is another frequent comorbidity in patients with psoriasis. Psoriasis alone can affect mental health; however, systemic inflammation through TNF may have an adverse effect on neurocognitive functions. Extensive data also suggest that depression increases the risk of cardiovascular morbidity and mortality [44]. The risk is already increased in mild depression such as vital exhaustion. The link between depression and atherosclerosis might be inflammation and endothelial dysfunction that are already present in patients with psoriasis [45].

It should be emphasized that most patients with psoriasis are being underdiagnosed and undertreated for these risk factors. These risk factors not only accelerate each other, they also have a negative impact on the effectiveness of systemic treatment for psoriasis [7]. We assume that, based on emerging knowledge, this risk will soon be individually estimated using specific imaging techniques for the detection of subclinical atherosclerosis and possibly biomarkers, as is also foreseen by recent studies that have begun to use imaging techniques for risk assessment. Particularly useful imaging techniques could be flow-mediated dilation (FMD) that determines endothelial (dys)function, carotid-femoral pulse wave velocity (PWV) that determines arterial stiffness, and augmentation index. All of these have been shown to correlate with cardiovascular risk in various populations of patients [46,47]. There are also studies that used the coronary artery calcium score and epicardial fat thickness as predictors of subclinical atherosclerosis [35]. The biomarkers that were used to estimate subclinical atherosclerosis in patients with psoriasis were serum myeloperoxidase, urinary orosomucoid, glycoprotein acetylation (GlycA), modified lipid oxidation, circulating growth differentiation factor-15 (GDF-15), and neutrophil-to-lymphocyte ratio (NLR) [35,48].

## 4. Common Ground for Targeting Psoriasis and Atherosclerosis at the Same Time

**Figure 2:**
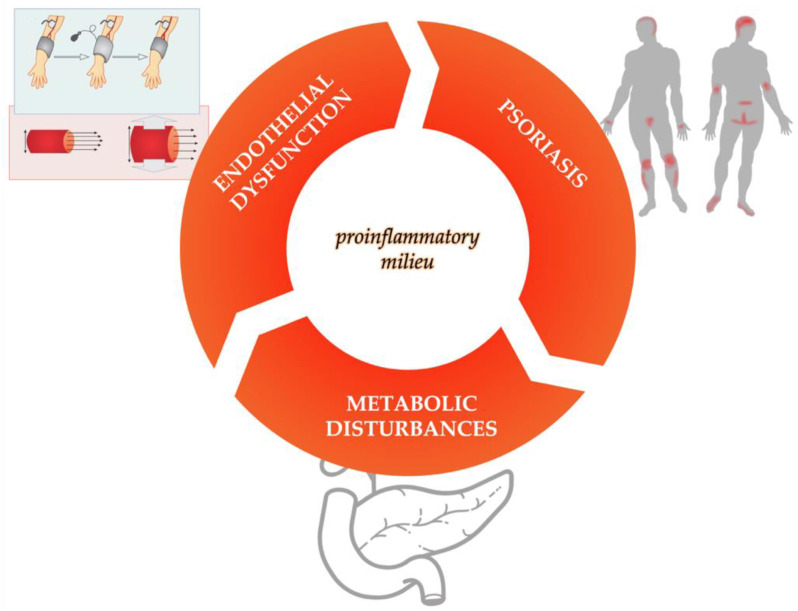
Psoriatic-metabolic-vascular continuum.

It is known that in patients with psoriasis, atherosclerosis is accelerated. However, the exact pathophysiological mechanisms have not yet been fully elucidated. As explained in more detail, there is a complex interaction between systemic inflammation, immune dysregulation, metabolic abnormalities, cardiovascular risk factors, and prothrombotic state leading to progressive cardiovascular damage and cardiovascular disease and forming the so-called “psoriatic-metabolic-vascular continuum” [49]. Therefore, it would be possible to target both psoriasis and atherosclerosis, since both diseases share at least some important common pathogenetic characteristics, as shown in Figure 2: 1. similar chronic systemic inflammation pathways with dysregulation of both the innate and adaptive immune systems are crucially involved in both [1]; 2. psoriatic plaques and atherosclerotic plaques share similar histopathologic characteristics with comparable dermal inflammation and leukocyte infiltration [7]; 3. psoriasis-induced inflammation promotes insulin resistance leading to endothelial dysfunction and deterioration of structural and functional characteristics of the arterial wall, both subclinical markers of atherosclerotic disease [1,7,9]. Based on these common mechanisms, it is assumed that effective treatment of one could target the other and vice versa. 

## 5. Anti-Psoriatic Treatment and Its Influence on Atherosclerosis

### 5.1. Traditional Systemic Agents

#### 5.1.1. Methotrexate

Methotrexate has been used for the treatment of moderate to severe psoriasis for decades. In psoriasis, methotrexate has been shown to exert anti-inflammatory effects by reducing the growth of leukocytes and triggering their apoptosis, decreasing the levels of pro-inflammatory levels of IL-1 and IL-6, increasing the levels of anti-inflammatory levels of IL-4 and IL-10 and decreasing the gene expression of Th1 cells [50].

In atherosclerosis, it has been shown to reduce cardiovascular risk, especially at low doses (5–25 mg weekly) [5]. On the other hand, the Cardiovascular Inflammation Reduction Trial (CIRT), in which low-dose methotrexate was evaluated for the prevention of atherosclerotic events, did not show fewer cardiovascular events compared to placebo-treated patients, leading the authors to conclude that stable atherosclerosis is characterized by low inflammatory burden (as in most of the patients recruited in the trial), while an increased inflammatory status is associated with instability of atherosclerotic plaques. Thus, the cardiovascular benefits of low-dose methotrexate may be only applicable to patients who have concomitant systemic inflammatory diseases in combination with atherosclerosis or who have unstable atherosclerosis [51]. Furthermore, a study by Krahel et al. also showed a beneficial effect of methotrexate in patients with psoriasis and atherosclerosis. In this study, patients with psoriasis were treated with methotrexate for three months and were found to have a significantly reduced level of proprotein convertase subtilisin/kexin type 9 (PCSK9) [50]. Since PCSK9 stimulates and maintains chronic inflammation and improves oxidative stress [52], this could be an important argument in the treatment of psoriasis with methotrexate.

#### 5.1.2. Retinoids (Acitretin)

In psoriasis, retinoids are used due to their anti-inflammatory effect and modulation of keratinocyte differentiation [53]. In animal studies, all-trans retinoic acid was shown to reduce IL-6, IL-12, and TNF-α levels, thus decreasing the inflammatory burden [54]. Furthermore, in one study, acitretin was shown to reduce leptin levels and decrease insulin resistance in psoriasis patients [55]. On the other hand, a statistically significant weight gain was observed. However, the authors posed an interesting hypothesis that acitretin could influence the structure of adipocytes, which would decrease adipokines production and cause a redistribution of fat from the visceral to the subcutaneous area [55]. This phenomenon is also observed with glitazones in which receptors interact with receptors of retinoids [56].

Regarding atherosclerosis, in a study comparing PCSK9 levels in monotherapy with methotrexate versus acitretin, acitretin treatment led to increased PCSK9 levels [50]. Dose-related retinoids-induced dyslipidemia could also influence the acceleration of atherosclerosis in patients with psoriasis [53]. However, these new insights into possible fat redistribution and adipokine production could provide some relief to clinicians that dyslipidemia triggered by retinoids might not contribute significantly to atherogenesis.

#### 5.1.3. Cyclosporine

Cyclosporine is an immunosuppressive drug that is used mainly in transplant recipients in whom atherosclerosis is markedly accelerated. This is mainly due to the side effects of cyclosporine on endothelial cells, smooth muscle cells, lipoprotein metabolism, and macrophages [57]. Cyclosporine works by inhibiting T-cell function, down-regulating ICAM-1 in keratinocytes and endothelial cells, which prevents the recruitment of inflammatory cells into the skin and decreasing vascular endothelial growth factor. Furthermore, it reduces the expression of Th17 pathway genes that decrease the levels of TNF-α, IL-17, IL-22, and IL-23p19 [58].

In psoriasis, the dose of cyclosporine is much lower than in transplant recipients; however, side effects are commonly observed, particularly hypertension and dyslipidemia [58,59]. Although it is an efficient therapeutic option in many patients with psoriasis, its use is not as common, as it can only be used as short-term therapy due to nephrotoxicity [58]. It is used mainly in the management of psoriasis crisis or as a bridge therapy [58].

In atherosclerosis, studies that measure direct cardiovascular risk in patients with psoriasis treated with cyclosporine are lacking. However, in one study, the levels of adiponectin and resistin of cyclosporine-treated patients with psoriasis increased significantly, while the weight gain of these patients was insignificant [60]. Therefore, cyclosporine could also contribute to insulin resistance and metabolic abnormalities.

#### 5.1.4. Fumaric Acid Esters

In psoriasis, fumaric acid esters represent an effective treatment option due to their yet precisely unknown immunomodulatory, anti-inflammatory, and antioxidant properties [61]. Fumaric acid esters were shown to decrease serum C-reactive protein (CRP) and increase the cardioprotective level of adiponectin [4]; however, more data are needed to evaluate additional possible cardioprotective effects.

### 5.2. Biologic Therapy

The biologic agents in the responders markedly reduce systemic inflammation, which is supposed to convey a reduction in the incidence of cardiovascular disease [62]. Furthermore, multiple cohort studies suggest that biologic agents may have an additional pleiotropic cardioprotective effect. However, there are still no conclusive data on the superiority of one biologic agent over others in terms of its cardioprotective effects. In a review by Kaushik et al., it is recommended that patients with coexisting cardiovascular risk factors receive TNF-α inhibitors due to most of the data and experience with their long-term use [5]. However, since newer biologic agents are used more frequently and are much more effective in reducing the onset of the disease, as suggested by improvement of PASI and systemic inflammation, new data are expected.

#### 5.2.1. Tumor Necrosis Factor-α Inhibitors (TNF-α Inhibitors) (Adalimumab, Etanercept, Infliximab, and Certolizumab)

In psoriasis, TNF-α inhibitors are an effective treatment option. They reduce inflammation by inhibiting pro-inflammatory cytokines (IL-1, IL-6, and IL-8), other pro-inflammatory molecules, and adhesion molecules (ICAM-1, P-selectin, and E-selectin) [63].

Regarding atherosclerosis, a meta-analysis showed that TNF-α inhibitors reduce cardiovascular events and mortality in patients with psoriasis [64]. Furthermore, TNF-α inhibitors also improve insulin sensitivity [65]. In the case of adalimumab, an improvement in endothelial function and carotid arterial stiffness was observed [66]. On the other hand, TNF-α inhibitors can cause increased body weight in patients with psoriasis. Therefore, patients treated with TNF-α inhibitors should be educated to maintain healthy body weight to maintain a lower cardiovascular risk [5].

#### 5.2.2. Anti-Interleukin-12/-23p40 Agents (Anti-IL-12/23p40) (Ustekinumab)

Inhibition of IL-12 results in inhibition of the Th1 response, whereas inhibition of IL-23 inhibits the Th17 response which results in decreased levels of TNF-α, IL-1β, IL-17A, and IL-6. In addition, it reduces vascular cell adhesion protein 1 (VCAM-1) [67]. These responses play an important role not only in psoriasis, but also in cardiovascular disease.

In psoriasis, anti-IL-12/23p40 is considered an effective treatment option [68].

Regarding atherosclerosis, a recent study that evaluated the impact of ustekinumab on aortic vascular inflammation showed a possible transient reduction in aortic vascular inflammation and a more durable reduction in aforementioned inflammatory cytokines associated with cardiovascular disease [67].

#### 5.2.3. Anti-IL-17 Agents (Ixekizumab, Secukinumab, and Brodalumab)

In psoriasis, head-to-head trials with other biologic agents (ustekinumab and etanercept) showed the superiority of anti-IL-17 agents in improving psoriasis [68].

Regarding atherosclerosis, the data are conflicting—some studies showed a pro-atherogenic role of IL-17, where its blockade reduced plaque vulnerability and cytokine expression [17]; in contrast to some other studies, where IL-17 showed an anti-atherogenic role [69]. IL-17A which is inhibited by anti-IL17 agents acts on endothelial cells where it stimulates the secretion of IL-1 and IL-6, upregulates inducible NOS, and activates matrix metalloproteinases (MMP) v [70]. Taking into account the main role of IL-17 in both atherosclerosis and psoriasis and its effectiveness in reducing inflammation, future studies will precisely determine the most likely protective role of anti-IL-17 agents in atherosclerosis.

#### 5.2.4. Anti-IL-23p19 Agents (Guselkumab, Risankizumab, and Tildrakizumab)

In psoriasis, anti-IL-23p19 agents are considered the most effective treatment option, since they can improve psoriasis by up to 100% [68]. Anti-IL-23p19 agents inhibit the development and propagation of Th17 cells, thus decreasing the levels of IL-17, IL-21, and IL-22 [35,68].

Regarding atherosclerosis, studies focusing on the cardiovascular effect of anti-IL-23p19 are currently lacking. An animal study proposed a possible harmful effect of inhibition of IL-23p19, since IL-23p19 was shown to protect against atherosclerosis by maintaining the intact intestinal barrier and regulating gut dysbiosis [71].

#### 5.2.5. JAK Inhibitors (Tofacitinib)

JAK inhibitors represent a new treatment option for patients with psoriasis. JAK inhibitors inhibit the signal pathway and gene transcription involved in the production of inflammatory cytokines [70]. Studies of JAK inhibitors for psoriasis are still limited. Among them, tofacitinib is the most studied JAK inhibitor in the treatment of psoriasis, while others have been examined in phase 2 trials with a PASI response less than or similar to that achieved with tofacitinib. Therefore, it is not likely that they will be further studied [70]. 

Regarding atherosclerosis, JAK inhibitors have been shown to have adverse effects on cardiovascular risk, trigger dyslipidemia, and increase the risk of thrombosis [68]. On the other hand, one study suggested that tofacitinib improved cardiovascular risk in patients with rheumatoid arthritis [72]. 

#### 5.2.6. Apremilast

Apremilast inhibits phosphodiesterase-4, increasing cyclic adenosine monophosphate (cAMP) which modulates the expression of pro- and anti-inflammatory mediators. This results in a reduction in TNF-α, IL-10, and IL-23 levels and a decrease in the expression of inducible NOS. Apremilast thus restores the balance between pro-inflammatory and anti-inflammatory signals [73]. In psoriasis, apremilast showed efficacy in the treatment of moderate to severe psoriasis [68]. 

Regarding atherosclerosis, in a clinical study 6 months of apremilast treatment in patients with psoriatic arthritis resulted in a decrease in body mass index, insulin resistance, inflammation, levels of apolipoprotein B, and improved endothelial dysfunction [74]. However, randomized controlled trials with apremilast did not show an increased risk of MACE [5]. More long-term studies are needed to evaluate its cardiovascular effect.

### 5.3. Rarely Used Systemic Agents

#### Colchicine

Colchicine is a potent anti-inflammatory medication that is rarely used in the treatment of psoriasis. However, it has been reported to be effective, especially in palmoplantar pustulosis and pustular psoriasis [75]. It inhibits the inflammasome of receptor protein 3 (NLRP3) of the nucleotide-binding domain, which then inhibits the production of IL-1, IL-6, and TNF-α [76]. 

Regarding atherosclerosis, its beneficial effect has already been established in large clinical trials showing a significantly lowered risk of ischemic cardiovascular events in patients with recent acute myocardial infarction [77] and in patients with chronic cardiac disease [78]. Furthermore, it has made its way into secondary prevention of atherosclerotic disease, particularly in high-risk patients [8]. This is explained by its inhibitory effect on endothelial cell dysfunction and inflammation, inhibition of smooth muscle cell proliferation, inhibition of platelet activation, and inhibition of macrophage chemotaxis, migration, and adhesion [79].

### 5.4. Summary of Antipsoriatic Treatment and Its Association with Atherosclerosis

In general, current evidence suggests that methotrexate, TNF-α inhibitors, anti-IL-12/23p40, and anti-IL-17 agents have a positive or at least neutral effect on cardiovascular health. Furthermore, one year of therapy with any of these biologic agents resulted in a reduction in the plaque burden of the coronary arteries [80]. On the other hand, the evidence for anti-IL-23p19 and JAK inhibitors is insufficient yet to provide any conclusions. Furthermore, Gisondi et al. also studied metabolic abnormalities of systemic therapies in psoriasis and concluded that in patients with psoriasis who have cardiometabolic comorbidities, acitretin and cyclosporine should be used with caution [81]. Mixed findings from studies that evaluate pre- and post-intervention cardiovascular risk are probably present due to differences in systemic inflammatory load, measured surrogate endpoints, usually small sample size, duration of study, and heterogeneity of inclusion criteria. More studies are expected to focus specifically on addressing cardiovascular risk.

## 6. Medications Used in the Prevention of Atherosclerotic Cardiovascular Disease That Have an Impact on Psoriasis

### 6.1. Statins

In the prevention of atherosclerosis, statins are the cornerstone of cardiovascular prevention [8]. Accepting the elevated cardiovascular risk in patients with psoriasis, the question arises of whether all patients with psoriasis should receive statin. According to the study by Mason et al., at least 60% of patients are eligible for statin therapy [82]. Furthermore, in addition to the LDL lowering effect, statins also have pleiotropic effects, including reduction of inflammation, angiogenesis, plaque thrombogenicity, endothelial dysfunction, and cellular migration [83]. 

Regarding psoriasis, some studies showed that statins reduced the risk of progression of psoriasis, while some reported worsening of psoriatic lesions [7]. A meta-analysis of randomized clinical trials showed an improvement in psoriasis in patients receiving statin therapy, particularly those with severe psoriatic disease [84]. A study showed an improvement in PASI and a reduced level of pro-inflammatory markers (IL-6 and high-sensitivity CRP (hsCRP)) after one month of therapy with rosuvastatin in patients with mild or moderate psoriasis. Therefore, the authors speculated that statins may have a wider spectrum of action in immune-mediated diseases in addition to their primary lipid-lowering action [85].

### 6.2. Antihypertensive Agents

The role of antihypertensive agents in the prevention of atherosclerosis is indisputable [8].

Regarding psoriasis, antihypertensive agents, especially beta-blockers, are known to cause possible exacerbations of psoriasis [7]. Furthermore, a large cohort study confirmed an increased risk of developing psoriasis in patients with arterial hypertension treated with beta-blockers for more than 6 years [86]. Beta-blockers can block beta-adrenergic receptors in the skin, causing a decrease in cAMP, which further triggers the pro-inflammatory state with stimulation of keratinocyte proliferation [86]. A recent review also showed that, in addition to beta-blockers, psoriasis can also be exacerbated in patients taking angiotensin-converting enzyme (ACE) inhibitors or angiotensin II receptor blockers (sartans) [87]. However, the precise mechanism by which ACE inhibitors or sartans could trigger psoriasis is still unknown [86]. To our knowledge, no data are yet available on the possible cardiovascular benefits of these drugs in patients with psoriasis, in addition to normalization of arterial blood pressure. However, arterial hypertension, which is one of the most prevalent cardiovascular risk factors, definitely requires treatment. First, by non-pharmacologic interventions and second (or concomitantly) with pharmacologic therapy. However, more studies are needed, preferably providing a recommendation on which antihypertensive drug would be most beneficial in patients with psoriasis with arterial hypertension. As ACE inhibitors have been proven to reduce major adverse cardiovascular events, they seem to be the logical drugs of choice [88].

### 6.3. Aspirin and Nonsteroidal Anti-Inflammatory Drugs 

In atherosclerosis, aspirin 75-100 mg daily is a cornerstone of secondary prevention due to its known antithrombotic effect [8]. A recent meta-analysis showed a lower risk of nonfatal myocardial infarction and ischemic stroke with aspirin but did not show a reduction in all-cause or cardiovascular mortality [89].

Regarding psoriasis, non-steroidal anti-inflammatory drugs (NSAIDs) have been reported to induce or exacerbate psoriasis. Furthermore, after long-term treatment with paracetamol and/or NSAIDs, the risk of psoriatic arthritis can increase. However, in the same study, aspirin did not show an increase the risk of psoriasis or psoriatic arthritis [90]. Despite its anti-inflammatory properties and the expectedly favorable cardiometabolic risk profile, it has not yet shown beneficial effects on psoriasis. 

### 6.4. Antihyperglycemic Agents

It appears that biguanides, thiazolidinediones, dipeptidyl peptidase-4 (DPP-4) inhibitors, and glucagon-like peptide-1 (GLP-1) receptor agonists, reduce the incidence of autoinflammatory and autoimmune disorders [3]. Furthermore, there are emerging data that suggest that several antihyperglycemic agents used in the treatment of type 2 diabetes have beneficial effects on psoriasis, including biguanides, sodium-glucose cotransporter-2 (SGLT-2) inhibitors, GLP-1 receptor agonists, DPP-4 inhibitors, and thiazolidinediones. These effects are explained by weight reduction, improved glycemic control, and direct effect on inflammation [7]. 

#### 6.4.1. Metformin

Metformin is the most widely used antidiabetic drug and remains one of the first-line therapies in the treatment of type 2 diabetes [91]. Regarding atherosclerosis, it exerts several cardiovascular protective effects in patients with diabetes, namely, it improves insulin resistance, reduces LDL and total cholesterol levels, improves endothelial function and function of vascular smooth muscle cells, inhibits IL-1β and inhibits cardiac remodeling [92,93].

Regarding psoriasis, metformin showed improvement in PASI and some metabolic parameters [91,94]. Furthermore, in patients with diabetes, it could reduce the risk of developing psoriasis [94]. In a randomized controlled trial, an improvement in psoriasis was observed in metformin-treated patients compared to placebo [95]. To accurately evaluate the role of metformin in psoriasis, more studies are needed. However, current evidence suggests that metformin is safe for use in psoriasis. Furthermore, with respect to its beneficial anti-inflammatory and other pleiotropic effects, it could prove especially useful as an additional treatment in patients with psoriasis with metabolic comorbidities, such as obesity, metabolic syndrome, and insulin resistance or diabetes [94].

#### 6.4.2. GLP-1 Receptor Agonists

GLP-1 receptor agonists are newer agents that are widely used for the treatment of type 2 diabetes. Their action extends beyond the treatment of type 2 diabetes [96]. Especially semaglutide has a beneficial effect on reducing body weight, both in patients with and without type 2 diabetes [92]. Furthermore, several studies have shown effective prevention of cardiovascular events and associated mortality by GLP-1 receptor agonists due to their cardioprotective effects, including decrease in blood pressure, improvement in dyslipidemia, increase in NO levels, and inhibition of adhesion and procoagulant factors [93,96]. Therefore, the guidelines of the European Society of Cardiology recommend GLP-1 receptor agonists as a possible first-line therapy for type 2 diabetes in patients with established atherosclerotic cardiovascular disease or in those at high or very high risk [97].

Regarding psoriasis, studies showed an improvement in psoriasis with GLP-1 receptor agonists by improving glycemic control, significant weight reduction, and possibly systemic anti-inflammatory effects [98,99,100]. It would be interesting to see whether these agents could also be beneficial due to their pleiotropic effects, particularly on inflammation, even in patients with psoriasis without diabetes. 

#### 6.4.3. Thiazolidinediones

Thiazolidinediones are used for glucose control in type 2 diabetes. Regarding atherosclerosis, they have been shown to have several beneficial pleiotropic effects on cardiovascular risk factors, namely improvement of insulin sensitivity, redistribution of fat from visceral to subcutaneous depot, reduction of blood pressure, and improvement of endothelial function [101]. 

Regarding psoriasis, when treated with pioglitazone, patients with psoriasis and metabolic syndrome had an improvement in the severity of psoriasis [7,95]. They exert anti-inflammatory effects, including reduced lymphocyte migration, reduced macrophage activation, modulation of monocytokine secretion, and decrease in IL-17 expression [102]. As mentioned above, thiazolidinediones cause a redistribution of fat from the visceral area to the subcutaneous area. This is important because visceral fat is an important contributor and prognostic marker to the risk and prognosis of coronary artery disease [103]. Since patients with psoriasis are commonly overweight with visceral fat accumulation [104], a redistribution of fat from the visceral to the subcutaneous region would certainly be beneficial to reduce their cardiovascular risk.

#### 6.4.4. SGLT-2 Inhibitors 

SGLT-2 inhibitors are known to have pleiotropic effects in the prevention of atherosclerotic cardiovascular disease by inhibiting vascular inflammation, reducing oxidative stress, improving endothelial dysfunction, preventing platelet activation, and reducing foam cell formation. Furthermore, they decrease the activation of the NLRP3 inflammasome, which is involved in the pathogenesis of both diseases, psoriasis, and atherosclerosis. This seems to be the class effect, although empagliflozin appears to be superior to others. Furthermore, a favorable reduction in body weight, blood pressure, and uric acid levels was observed in patients treated with SGLT-2 inhibitors [105].

Regarding psoriasis, to our knowledge, there are yet no studies that specifically address the effects of SGLT-2 inhibitors on psoriasis or cardiovascular risk in patients with psoriasis. However, depending on the aforementioned pleiotropic effects of SGLT-2 inhibitors, including their anti-inflammatory effect, future studies are likely to also demonstrate their beneficial role in psoriasis.

### 6.5. Inhibitors of IL-1β (Anakinra, Canakinumab, and Rilonacept)

IL-1 family cytokines are products of the inflammasome and exert important vascular and systemic inflammatory effects that promote atherogenesis. Therefore, inhibition of IL-1 with IL-1β inhibitors can improve cardiovascular outcomes by inhibiting the formation of atheromatous lesions, decreasing vascular inflammation, stabilizing atherosclerotic plaque, and preventing harmful cardiac remodeling [106]. A recent trial confirmed the reduction of recurrent cardiovascular events in patients with previous acute myocardial infarction [107].

Regarding psoriasis, a study in which IL-1 inhibition was used in plaque psoriasis showed a decrease in PASI of only 13%, which was lower than that obtained with other biologic agents (anti-TNF, anti-IL-17, and anti-IL-12/-23p40) [102]. However, the expression of auto-inflammatory components probably differs between the subtypes of psoriasis, the phenotypes of psoriasis, the inflammation of psoriasis, and patients with psoriasis. Therefore, the role of inhibition of IL-1 in psoriasis-induced cardiovascular risk remains to be elucidated.

### 6.6. Summary of Preventive Anti-Atherosclerotic Agents and Their Associations with Psoriasis

Evidence from large cardiovascular outcome trials, such as the CANTOS, CIRT, Colchicine Cardiovascular Outcomes Trial (COLCOT), and Low Dose Colchicine 2 (LoDoCo2), highlight how important inflammation targeting is in atherosclerotic cardiovascular disease [108]. Furthermore, in the case of concomitant systemic inflammatory diseases such as psoriasis, the atherosclerotic burden is even higher; therefore, inflammation should be markedly reduced by agents that address both psoriasis and atherosclerosis.

Depending on the data on which medications have been shown to have the greatest cardiovascular benefit in atherosclerosis, we can hypothesize that future studies will also show their beneficial impact in reducing the atherosclerotic load in psoriasis. In our opinion, such promising agents are high potency statins (particularly rosuvastatin), metformin, SGLT-2 inhibitors (with empagliflozin’s probable superiority), GLP-1 receptor agonists (particularly emerging with great cardiovascular benefits such as semaglutide and liraglutide), anti-IL-17, anti-IL-23, and agents that inhibit IL-1β. Possible pleiotropic effects of other treatments (antihypertensive therapy, especially ACE inhibitors/sartans and other antihyperglycemic agents) will probably also be evaluated in the future. It is surely important to emphasize that with any adjuvant therapy (cardiometabolic or systemic anti-inflammatory) therapy, there should be a precise evaluation of the risk-benefit ratio. 

Table 1 summarizes all the described agents and there possible anti-psoriatic and anti-atherosclerotic effects, also in regard to clinical outcomes.

## 7. Conclusions

Future perspectives in the treatment of psoriasis could be adjuvant treatment with medications that target psoriasis-driven atherosclerosis. This hypothesis is based on shared pathways, including endothelial dysfunction, oxidative stress, arterial and cardiac remodeling, increased angiogenesis, prothrombotic state, monocyte and neutrophil recruitment, and T cell activation. The complex interplay among all those mechanisms could be approached, at least in theory, by different drugs, and consequently, a decrease in the rate of primary pathological processes would be observed. Furthermore, both diseases are influenced by the metabolic syndrome and are improved by correcting its features. Therefore, it seems reasonable that further studies of psoriasis therapy (especially biologic agents) include a global pre- and post-interventional cardiometabolic risk assessment. Furthermore, further studies should also assess the impact of adjuvant cardiovascular prevention agents, particularly statins, metformin, SGLT-2 inhibitors, and GLP-1 receptor agonists, on primary endpoints of psoriasis (PASI) and surrogate markers of atherosclerotic cardiovascular disease (e.g., endothelial dysfunction). Furthermore, due to the conflicting results of many studies, inflammatory status should be evaluated prior to starting treatment. 

Taking into account all the data, a physician who treats patients with psoriasis should therefore consider several treatment goals. To reduce the burden of primary disease by addressing psoriasis per se; to reduce the burden of inflammation, by effective treatment of psoriasis and screening and treating other inflammatory comorbidities (psoriatic arthritis, nonalcoholic fatty liver disease, and chronic inflammatory bowel disease); to reduce cardiometabolic risk, by screening for and addressing all cardiovascular risk. Risk factors should be evaluated at each outpatient visit and addressed quickly and appropriately. The screening for depression should also be performed immediately. Even mild depression (such as vital exhaustion) increases cardiovascular morbidity. Referral to a psychologist/psychiatrist should not be delayed.

On the other hand, a patient should be educated and encouraged about non-pharmacological treatment: physical activity, reduction of body weight and/or maintaining normal weight, having a healthy and balanced diet (without or markedly reduced intake of saturated fatty acids, red meat, noncomplex sugars, or alcohol), reduction of emotional stress, and cessation of smoking. 

In summary, in the era of groundbreaking progress in the effective treatment of skin psoriasis with biologic therapy, the prevention of atherosclerosis as the most frequent cause of morbidity and mortality in this population is slowly gaining momentum. Therefore, we assume that further studies with the spectrum of therapy will provide an in-depth assessment of cardiovascular risk and/or subclinical atherosclerosis before and after the evaluation, allowing a better holistic treatment approach in this vulnerable population.

## Figures and Tables

**Table 1:** Summary of agents used in the treatment of psoriasis and in the prevention/treatment of atherosclerotic cardiovascular disease and their anti-psoriatic and anti-atherosclerotic effects. IL—interleukin, PASI—Psoriasis Area Severity Index; (hs)CRP—(high sensitivity) C-reactive protein; PCSK9—proprotein convertase subtilisin/kexin type 9; TNF-α—tumor necrosis factor-α; MACE—major adverse cardiovascular events; ICAM—intercellular adhesion molecule; VEGF—vascular endothelial growth factor; VCAM—vascular cell adhesion molecule; NOS—nitric oxide synthase; MMP—matrix metalloproteinase; cAMP—cyclic adenosine monophosphate; JAK—Janus kinase, GLP-1—glucagon-like peptide-1, and SGLT-2—sodium-glucose cotransporter-2.

		Agent(s)	Anti-Psoriatic Effects	Clinical Outcomes in Psoriasis	Anti-Atherosclerotic Effects	Clinical Outcomes in Atherosclerosis
Psoriasis treatment	Traditional systemic agents	Methotrexate	Anti-inflammatory action (↓ leukocytes, ↓ IL-1 and IL-6, ↑ IL-4 and IL-10, ↓ Th1 cells) [50]	Decrease in PASI [50,51,109,110]	Anti-inflammatory action (↓ IL-6, CRP and IL-1β) [51,108] ↓ PCSK9 level [50]	Reduction of cardiovascular risk if elevated inflammatory burden [50,51,109,110]
		Retinoids	Anti-inflammatory action (↓ IL-6, IL-12, TNF-α) [53,54,55,56] Modulation of keratinocyte differentiation [53]	Decrease in PASI [53,58]	Reduction in leptin levels [55] Improvement of insulin resistance [55]; Redistribution of fat from visceral to subcutaneous area [55]	Unknown effect on MACE
		Cyclosporine	Inhibition of T-cell function [58]; Down-regulation of ICAM-1 on keratinocytes and endothelial cells [58] Reduction of expression of Th17 pathway genes [58] ↓ TNF-α, IL-17, IL-22, IL-23p19 [58] ↓VEGF [58]	Decrease in PASI; useful in the management of psoriatic crises or as a bridge therapy [58,59]	Increase adiponectin and resistin [60]	Unknown effect on MACE
		Fumaric acid esters	Immunomodulatory, anti-inflammatory and antioxidant effects that are not yet completely understood [4,61]	Decrease in PASI [4]	↓ CRP and ↑ adiponectin levels [4]	Unknown effect on MACE
	Biologic agents	TNF-α inhibitors	Anti-inflammatory action (↓ IL-1, IL-6, IL-8, ICAM-1, P-selectin and E-selectin [5,63]	Decrease in PASI [5,63]	Improvement in insulin sensitivity [65] Adalimumab improves endothelial function and carotid arterial stiffness [64,66]	Reduction of cardiovascular events and mortality [64] Reduction of coronary artery plaque burden after one year of treatment [80]
		Anti-IL-12/23p40 agents	Inhibition of Th1 and Th17 response [68] ↓ TNF-α, IL-1β, IL-17A, IL-6, VCAM-1 [67]	Decrease in PASI [68]	Transient decrease in aortic vascular inflammation [67] ↓ TNF-α, IL-1β, IL-17A, IL-6, VCAM-1 [67]	Reduction of coronary artery plaque burden after one year of treatment [80]
		Anti-IL-17 agents	Inhibition of IL-17A [68] Reduction of recruitment of Th17 cells, neutrophils, and dendritic cells to the skin [111]	Decrease in PASI; superior to anti-TNF-α and anti-IL-12/23p40 [68]	↑ IL-1 and IL-6 secretion [111] up-regulation of inducible NOS [111] inhibition of MMPs in endothelial cells [111,112] Induction of pro-atherogenic gut microbiota that could have a pro-atherogenic effect [71]	Reduction of coronary artery plaque burden after one year of treatment [80] No significant increase in MACE
		Anti-IL-23p19 agents	Inhibition of Th17 cell development and propagation [68] ↓ IL-17, IL-21 and IL-22 [68]	Decrease in PASI [68]		Unknown effect on MACE
	Others	JAK inhibitors	Inhibition of signal pathway and gene transcription involved in inflammatory cytokine production [68,70]	Decrease in PASI [68,70]	Reduction of carotid intima-media thickness [72] Limitation of vascular damage [72] ↑ fasting total cholesterol levels [72]	Improved cardiovascular risk in one study [72]
		Apremilast	↑ cAMP resulting in immunomodulation [73] ↓TNF-α, IL-10 and IL-23 [73] ↓ expression of inducible NOS [68,73]	Decrease in PASI [68,70]	Decrease in insulin resistance, levels of apolipoprotein B, body mass index [74] Improvement of endothelial function [74]	No increased risk for MACE [5]
	Rarely used systemic agent	Colchicine	Inhibition of inflammasome [75] ↓ IL-1, IL-6, TNF-α [75,76]	Improvement of pustular psoriasis and palmoplantar pustulosis [75]	Inhibition of endothelial cell dysfunction and inflammation [79] Inhibition of smooth muscle cell proliferation and migration [79] Inhibition macrophage chemotaxis, migration, and adhesion [79] Inhibition of platelet activation [8,77,78,79]	Reduction in MACE [8,77,78] Recommendation in secondary prevention in patients with high cardiovascular risk [8]
Cardiometabolic treatment	Anti-atherosclerotic treatment	Statins	Reduction of inflammation and angiogenesis [84,85] ↓ IL-6 and hsCRP [84,85]	Decrease in PASI [84]	↓ LDL [83] Improvement of endothelial dysfunction [83] Inhibition of cellular migration [83] Inhibition of plaque thrombogenicity [8,83]	Reduction in MACE [8]
		Antihypertensive agents	Beta-blockers can block beta-adrenergic receptors in skin resulting in ↓cAMP which triggers pro-inflammatory state with stimulation of keratinocyte proliferation; With respect to ACE inhibitors, probably complex pathways are involved which are yet unknown.	Might trigger psoriasis (especially beta-blockers, less likely ACE inhibitors) Unknown effect on PASI	Suppression of vasoconstriction, smooth muscle proliferation, connective tissue synthesis, and chemotaxis of monocytes by angiotensin II [8,88]	Reduction in MACE [8]
		Aspirin	Insufficient data	Possible induction or exacerbation of psoriasis—insufficient data	Antithrombotic effect [8]	Used in the secondary prevention of atherosclerosis; Lowers the risk of nonfatal myocardial infarction and ischemic stroke [89] No reduction in all-cause or cardiovascular mortality [8,89]
	Antihyperglicemic agents	Metformin	Anti-inflammatory action [95,113] ↓ IL-1β [93,94,97,98]	Possible decrease in PASI [95]	Improvement of insulin resistance [113] ↓ LDL and total cholesterol [113] Improvement of endothelial dysfunction [113] Improvement of function of vascular smooth muscle cells [113] Inhibition of cardiac remodeling [113]	Reduction in MACE in patients with pre-diabetes [114]
		GLP-1 receptor agonists	Anti-inflammatory action by ↓ IL-17 [98,99,100]	Decrease in PASI [98,99,100]	↓ blood pressure [93] ↓ body weight [93] Improvement of dyslipidemia [93] ↑ NO [93] Inhibits of adhesion and procoagulant factors [96,97]	Reduction of MACE in patients with type 2 diabetes [103]
		Thiazolidinediones	Anti-inflammatory effects: Reduction of lymphocyte migration [115] Reduction of macrophage activation [115] Decrease in IL-17 expression [115] Modulation of monocyte cytokine secretion [7,95,115]	Increase in PASI [7,95]	Improvement of insulin sensitivity [101] Redistribution of fat from visceral to subcutaneous depot [101] ↓ blood pressure [101] Improvement of endothelial function [101]	Reduction of MACE in patients with type 2 diabetes [101,107]
		SGLT-2 inhibitors	Unknown—no data	Insufficient data	Inhibition of vascular inflammation [116] Reduction of oxidative stress [116] Improvement of endothelial dysfunction [116] Prevention of platelet activation [116] Reduction of foam cell formation [116] Decrease in inflammasome activation [105,116]	Reduction of MACE in patients with type 2 diabetes [105]
	Other	IL-1β inhibitors	Inhibition of systemic inflammation [102]	Mild decrease in PASI [102]	Inhibition of the formation of atheromatous lesions [112] Decrease in vascular inflammation [112] Plaque stabilization [112] Prevention of harmful cardiac remodeling by inhibition of MMPs [107]	Reduction of recurrent cardiovascular events in patients with previous acute myocardial infarction [107]

## Data Availability

Not applicable.
